# SOCIAL DETERMINANTS OF HEALTH UNDERLIE RACIAL/ETHNIC DISPARITIES IN PSYCHOLOGICAL HEALTH AND WELL-BEING

**DOI:** 10.1093/geroni/igac059.1940

**Published:** 2022-12-20

**Authors:** Dylan Jester, Jordan Kohn, Lize Tibirica, Michael Thomas, Lauren Brown, James Murphy, Dilip Jeste

**Affiliations:** University of California San Diego, La Jolla, California, United States; University of California San Diego, La Jolla, California, United States; University of California, San Diego, San Diego, California, United States; Colorado State University, Fort Collins, Colorado, United States; San Diego State University School of Public Health, San Diego, California, United States; University of California San Diego, La Jolla, California, United States; University of California San Diego, La Jolla, California, United States

## Abstract

This study sought to determine the impact of selected social determinants of health on psychological health and well-being (defined as depression, cognition, self-rated health) among Black and Hispanic/Latinx adults relative to White adults aged 51 to 89. We measured disparities in depression, cognition, and self-rated health among 2,306 Non-Hispanic/Latinx Black, 1,593 Hispanic/Latinx, and 7,244 Non-Hispanic/Latinx White adults from the Health and Retirement Study (n=11,143). Blinder-Oaxaca decomposition was used to examine whether differences in selected social determinants of health explained a larger share of the disparities than age, sex, measures of health, health behaviors, and healthcare utilization. Selected social determinants included education, parents’ education, number of years worked, marital status, veteran status, geographic residence, nativity status, income, and insurance coverage. Black and Hispanic/Latinx adults reported worse depression, cognition, and self-rated health compared to White adults. Selected social determinants of health explained a larger proportion of the Black-White disparities in depression (51%), cognition (39%), and self-rated health (37%) than did age, sex, measures of health, health behaviors, and healthcare utilization. Social determinants of health explained a larger proportion of the Hispanic/Latinx-White disparity in cognition (76%) and self-rated health (75%), but age and functional status primarily drove the disparity in depression (28%). Education, parents’ education, years worked, income, and insurance parity were social determinants associated with the disparities.In conclusion, differences in social determinants of health underlie racial/ethnic disparities in depression, cognition, and self-rated health among older adults. Education, income, number of years worked, and insurance parity are key social determinants of health.

